# Can a social media intervention improve online communication about suicide? A feasibility study examining the acceptability and potential impact of the #chatsafe campaign

**DOI:** 10.1371/journal.pone.0253278

**Published:** 2021-06-15

**Authors:** Louise La Sala, Zoe Teh, Michelle Lamblin, Gowri Rajaram, Simon Rice, Nicole T. M. Hill, Pinar Thorn, Karolina Krysinska, Jo Robinson

**Affiliations:** 1 Orygen, Parkville, Victoria, Australia; 2 Centre for Youth Mental Health, The University of Melbourne, Parkville, Victoria, Australia; 3 Telethon Kids Institute, Perth, Western Australia, Australia; 4 Centre for Mental Health, The Melbourne School of Population and Global Health, University of Melbourne, Parkville, Victoria, Australia; Universidad Publica de Navarra, SPAIN

## Abstract

There is a need for effective and youth-friendly approaches to suicide prevention, and social media presents a unique opportunity to reach young people. Although there is some evidence to support the delivery of population-wide suicide prevention campaigns, little is known about their capacity to change behaviour, particularly among young people and in the context of social media. Even less is known about the safety and feasibility of using social media for the purpose of suicide prevention. Based on the #chatsafe guidelines, this study examines the acceptability, safety and feasibility of a co-designed social media campaign. It also examines its impact on young people’s willingness to intervene against suicide and their perceived self-efficacy, confidence and safety when communicating on social media platforms about suicide. A sample of 189 young people aged 16–25 years completed three questionnaires across a 20-week period (4 weeks pre-intervention, immediately post-intervention, and at 4-week follow up). The intervention took the form of a 12-week social media campaign delivered to participants via direct message. Participants reported finding the intervention acceptable and they also reported improvements in their willingness to intervene against suicide, and their perceived self-efficacy, confidence and safety when communicating on social media about suicide. Findings from this study present a promising picture for the acceptability and potential impact of a universal suicide prevention campaign delivered through social media, and suggest that it can be safe to utilize social media for the purpose of suicide prevention.

## Introduction

Suicide is the leading cause of death among young Australians and the second worldwide, with rates steadily increasing over the past decade [1–3]. Although young people who die by suicide frequently experience mental ill-health, many are reluctant to seek professional help and are not in contact with services at the time of their death [4]. In light of this, more effective, youth-friendly, and community-based suicide prevention initiatives are required.

As social media use increases among young people [5, 6] a growing body of literature points to the potential for online interventions to improve mental health outcomes [7–11], including in suicide prevention [12–16]. These studies have identified that social media can provide an accessible and acceptable forum for young people to communicate about suicide, seek support for themselves and also support others [8, 16–18]. These interventions also have the potential to facilitate access to specialist care [19, 20]. There are however downsides to using social media to communicate about suicide. For example, concerns exist regarding the potential for certain types of content (e.g., graphic images of suicide methods) to cause distress or harm to others [21, 22], and in some cases the spreading of suicide-related information via social media has been thought to contribute to the development of suicide clusters [21]. Despite these concerns, social media remains popular with young people [23], and therefore interventions that can better equip them to communicate about suicide safely on these platforms are required [16, 17].

Guidelines to support safe communication about suicide by mainstream media have become a widely accepted suicide prevention strategy in many countries including Australia [24–27], and when adopted by journalists, they appear to be linked to improvements in the quality of media reporting and a reduction in suicide rates [28]. However, because of its dynamic and interactive nature, young people communicate about suicide on social media in fundamentally different ways to the ways in which mainstream media operates [16, 29, 30]. For this reason existing guidelines are unlikely to have much traction with young people; nor are they necessarily transferable to social media platforms.

In response to this, we developed the #chatsafe guidelines which were specifically designed with both young people and social media platforms in mind (see www.orygen.org/chatsafe/). The guidelines were developed using the Delphi expert consensus method and include information on how to safely post about suicidal thoughts or experiences, engage with suicide content, respond to someone affected by or at risk of suicide, and how to manage memorial pages and closed groups [29]. We then worked in partnership with young people from across Australia to co-design a social media campaign to help disseminate the guidelines and facilitate their uptake [30]. The #chatsafe campaign was rolled out across three states and two territories in Australia between September 2019 and January 2020.

Previous campaigns targeting physical health outcomes in young people have been shown to be effective, for example in reducing sedentary behaviour and smoking, as well as improvements in sexual health [31]. Factors believed to facilitate behaviour change include evoking emotional responses that assist learning, depicting relevant and meaningful stories through familiar characters, and involving young people themselves in the creation and delivery of the campaign [32]. With this in mind, the delivery of population-wide campaigns has gained attention as a potentially effective suicide prevention strategy. Whilst some evidence exists to suggest that they can improve outcomes such as knowledge, awareness, and attitudes toward help-seeking [6, 33–38], there is a lack of evidence to support their capacity to change behaviour, particularly among young people and in the context of social media. There is also no evidence to date regarding the acceptability, safety, or feasibility of conducting and testing the impact of, suicide prevention campaigns on social media platforms.

Thus, the aims of this study were to examine the acceptability and safety of the #chatsafe campaign, and the feasibility of delivering and testing this intervention entirely via social media. Additional aims were to examine the impact of the intervention on young people’s willingness to intervene against suicide (e.g., feeling confident in their ability to discuss suicide with someone who is suicidal), as well as their perceived self-efficacy, confidence and safety when communicating about suicide on social media platforms.

## Methods

### Study design

**Fig 1:**
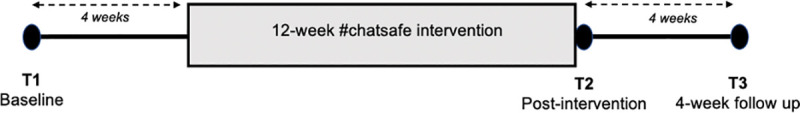
Timeline of study and delivery of #chatsafe intervention.

This study adheres to the Template for Intervention Description and Replication (TIDieR) checklist [39]. The study employed a single group pre-test/ post-test survey design with a 12-week intervention period. Participants completed self-assessments at three timepoints (baseline—T1, post-intervention—T2, and 4-weeks post-intervention—T3). See Fig 1.

The study was conducted by researchers based in Melbourne, Australia. It received approval from the University of Melbourne Human Research and Ethics Committee (ID:1954623).

### Participants and recruitment

Young people were eligible for inclusion in the study if they were aged between 16 and 25 inclusive, in line with the youth participation policy at the organisation where the research was conducted. Participants aged 16 and 17 years were determined by the ethics committee to be mature minors who were able to provide informed consent to participate. Eligibility criteria also included that they lived in Victoria, New South Wales or Tasmania, Australia, had not already read the #chatsafe guidelines or been exposed to the campaign, and endorsed any of the following: 1) had used social media to talk about suicide; 2) managed, or were part of, a suicide discussion group; 3) had viewed suicide-related content on social media; and/or 4) had wanted to talk about suicide on social media but did not feel equipped to do so. Study requirements also asked participants to provide their social media handles for Facebook, Instagram, Snapchat, Twitter or Tumblr.

Participants were recruited over a three-month period (September 2019 –December 2019) via social media advertising on Facebook, Instagram, Snapchat, YouTube and Twitter. Individuals who clicked into the online survey were screened for eligibility and those eligible for inclusion were asked to provide consent. Participants completed the baseline assessment immediately after providing consent (T1), the second assessment at the end of the 12-week intervention (T2), and the third assessment four-weeks after the intervention concluded (T3). All participants were reimbursed AUD$30 per assessment.

As seen in Fig 1, there was a four-week gap between the baseline assessment and commencement of the intervention (i.e., delivery of the first piece of #chatsafe content), and another four weeks between T2 and T3, meaning the study period was 20 weeks.

### The #chatsafe intervention

**Fig 2:**
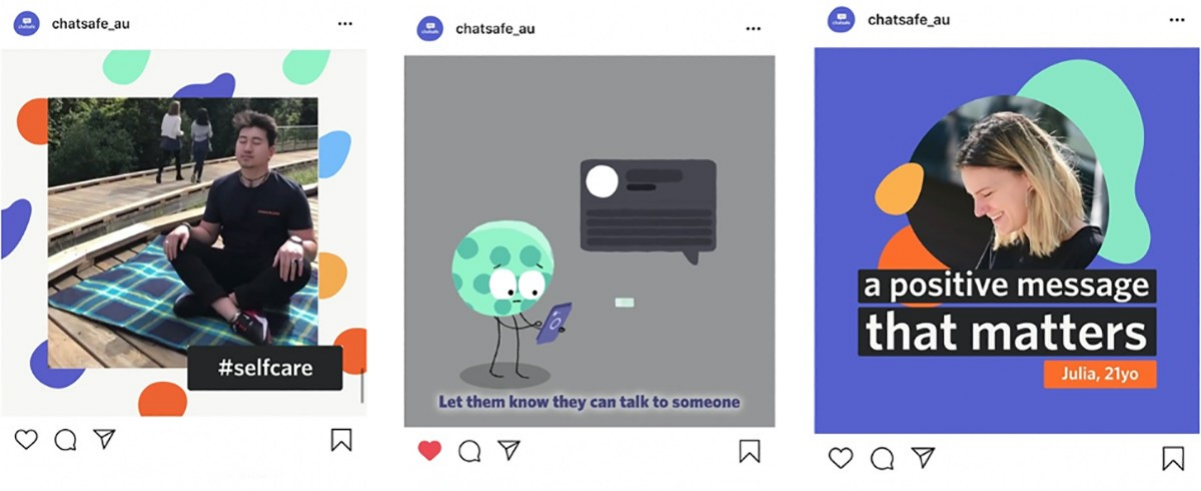
Examples of social media content shared during the #chatsafe campaign. Image 1: A still image of a short video (with no audio) depicting a young person “taking a break”. Image 2: A still image of an animation video that discusses how to support a friend who might be suicidal. Image 3: A photo and quote by a young person.

The intervention was delivered to participants once a week for 12 weeks via a direct message sent by one of the research team (LLS or ZT) to a social media account of the participants’ choice. Each message included a link to one piece of social media content (either a short video, animation or static image) that was hosted on the #chatsafe Instagram page (www.instagram.com/chatsafe_au). Each social media post directly mapped onto one of the themes from the guidelines e.g., safe language to use when talking about suicide. In order to avoid over-exposure to content relating specifically to suicide, every alternate week the content had a self-care theme. Self-care content included information about digital literacy and on- and off-line wellbeing. Content themes and the delivery schedule are described in Table 1 and examples of the campaign content are presented in Fig 2.

**Table 1:** Delivery schedule, content theme and content type for each week of the #chatsafe social media campaign.

Week	Content theme	Content type
1	General introduction to #chatsafe	Animation
2	Self-care	Animation
3	Responding to someone who might be suicidal	Text tile
4	Self-care	Video (no audio)
5	Safe posting on social media	Text tile
6	Self-care	Video (with audio)
7	Before you post, pause and reflect	Animation
8	Self-care	Video (with audio)
9	Remembering someone who has died by suicide	Animation
10	Self-care	Photo (and quote)
11	Dealing with harmful content	Text tile
12	Self-care	Photos

As this study ran alongside the national campaign, participants were able to view the wider campaign content posted on social media in addition to the individual pieces of content sent to them via direct message. This also meant that they could engage with the content as much or as little as they wished.

### Outcomes and outcome measures

All questionnaires were completed online via Qualtrics. Demographic information was collected at baseline using a 10-item purpose-designed questionnaire assessing age, nationality, Aboriginal or Torres Strait Islander identity, gender identity, and state of residence. Time spent on social media at baseline was measured using the Patterns of Social Media Use Questionnaire [6].

Each standardised measure (i.e., willingness to intervene against suicide online, perceived self-efficacy, and confidence and safety when communicating online about suicide) was measured at baseline (T1), T2 and T3. In addition to this, a short emoji scale measuring acceptability and safety accompanied the #chatsafe campaign content sent to participants each week. Acceptability data was also collected through a series of evaluation questions administered at T2 (see Fig 1).

#### Acceptability

**Fig 3:**
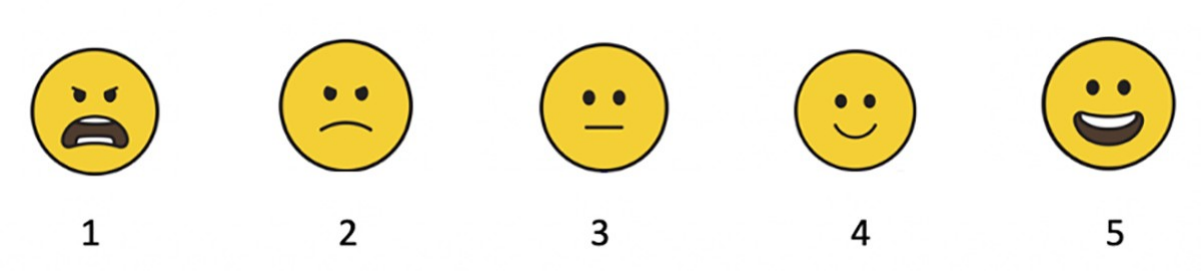
Evaluation emoji rating scale with 1 coded as most negative/distressed and 5 coded as most positive/happy.

Acceptability was assessed in the following ways. First, a purpose-designed three-item acceptability questionnaire was sent with each piece of weekly content. This short momentary-assessment asked participants: 1) What did you think about the campaign content this week?; 2) Would you share this week’s campaign content with your contacts on social media?; and 3) How did the campaign content you received today make you feel?. Each question was presented on a 5-point scale comprising a series of emojis depicting different mood states (see Fig 3). Participants also had the option to ‘snooze’ the delivery of the campaign, which would suspend delivery of the content for one week. Participants who selected response options 1 or 2 (see Fig 3) to the question relating to how the content made them feel were assessed as potentially showing signs of distress and asked if they would like to snooze the content for one week or withdraw from the study. Participants had to confirm that they would like to continue receiving #chatsafe content the following week to remain in the study.

Second, a series of evaluation questions were administered at T2. These questions asked participants if they found the content helpful, if it increased their confidence to talk safely online about suicide, if they thought it would be helpful for others, and if they felt that the campaign had any negative effects on them or if they thought it would have a negative effect on others.

#### Safety

Detailed safety procedures were developed. This included the establishment of an independent Safety Monitoring Committee to oversee study safety and conduct, comprising a clinical psychologist, an external subject-matter expert and an organizational operations manager, all of whom have extensive experience conducting clinical trials with young people.

Participant safety was assessed daily by monitoring the #chatsafe social media accounts for any messages or comments that indicated distress, and by monitoring the weekly survey responses for participants who snoozed or withdrew from the study (who were contacted within 24 hours). Contact details of relevant support services such as eheadspace and Kids Helpline were included in all study materials.

In addition, any adverse events (AEs) and serious adverse events (SAEs) that were brought to our attention were recorded. AEs were defined as any untoward or adverse effect, whether or not related to the study (e.g., comments that expressed suicidal ideation). SAEs were defined as an event that resulted in death and/or was immediately life threatening and/or required hospitalization [40]. All adverse events were monitored and recorded by a member of the study team (KK) with oversight from the study psychologist (SR). They were then reported to the Safety Monitoring Committee who determined whether or not the event was considered attributable to the #chatsafe intervention, if it could be appropriately dealt with by the existing safety protocols, or if the intervention needed to be withdrawn or suspended.

#### Feasibility

Criteria relating to feasibility were based on participant recruitment, attrition, and the reach of the broader campaign (including the overall number of impressions, and the number of times the post was ‘liked’ or viewed). Social media metrics were recorded and analysed by our digital design partners, Portable. As this was an exploratory study, no a priori social media metrics were set.

#### Willingness to intervene against suicide online

Participants’ perceived ability and intention to intervene against suicide were measured using two adapted subscales of the Willingness to Intervene Against Suicide Questionnaire [41]. The Perceived Behavioral Control subscale comprised 20 Likert-type items and assessed the participant’s confidence and belief in their ability to intervene with someone who might be at risk of suicide. The Intent to Intervene subscale comprised 22 items and assessed the participant’s ability to recognize the need for action, encourage help-seeking, and connect the suicidal person with resources or services. Items were scored on a 5-point scale (1 = Strongly disagree to 5 = Strongly agree), and composite scores for the Perceived Behavioral Control and Intent scales ranged from 20 to 100 and 22 to 110, respectively. Both subscales were adapted to remove the emphasis of seeking help in a college campus setting (e.g., locate someone on campus for the suicidal person to talk to), and increase emphasis on seeking information online (e.g., I would feel comfortable seeking information from a credible source online). Excellent reliability was observed for both the Perceived Behavioral Control (Cronbach’s α = 0.92) and the Intent to Intervene (Cronbach’s α = 0.90) subscales.

#### Perceived self-efficacy, confidence and safety when communicating online about suicide

Perceived self-efficacy was measured using an adapted version of the Internet Self-Efficacy scale [42], which comprised 17 items on a 7-point Likert scale (1 = Totally not confident to 7 = Completely confident), with composite scores ranging from 17 to 119. This assessed participants’ levels of confidence on reactive/generative, differentiation, organization, communication, and search self-efficacy, with higher scores indicating a higher level of internet self-efficacy. Reliability ranged from acceptable to excellent for the five domains (reactive/generative: α = 0.85; differentiation: α = 0.91; organization: α = 0.86; communication: α = 0.73; search: α = 0.82).

These five domains are categorized into three general levels of self-efficacy: high, medium and low. Domains with high levels of self-efficacy include communication (navigating social networking sites) and search (using advanced search engines) self-efficacy. Domains with medium levels of self-efficacy include organization (organizing information that may already be partially structured by the platform in use) and differentiation (participants’ willingness to follow hyperlinks in goal-oriented tasks). The domain with the lowest level of self-efficacy is a combination of reactive problem-solving (participant’s perceived ability to react and solve problems online) and generative self-efficacy (participants’ perceived ability to contribute unique information online).

Perceived safety was measured using an adapted version of the Perceived Safety Questionnaire [43]. This measure has previously been used to assess risk perception, including perceived safety, agency, coping and resolution online with a sample of young people, however not with reference to suicide-related content. Adaptations included making it specific to suicide-related content online (i.e., creating a post about suicide, viewing suicide-related information, or sharing suicide-related information on social media). This measure asked participants for information relating to the frequency and type of suicide-related content that was seen on social media throughout the study period.

### Data analysis

Descriptive statistics were used to assess acceptability, safety and feasibility. Acceptability was measured based on responses to the T2 evaluation questions. Safety was monitored daily and assessed weekly by responses to the Acceptability questionnaire. Ordinal logistic regression was used to ascertain whether participant characteristics were associated with weekly evaluations of campaign content. Participant characteristics considered were age, gender, sexual orientation, Aboriginal and/or Torres Strait Islander status, nationality, language spoken at home and current student status. Feasibility metrics were gathered based on recruitment, retention, and attrition, as well as social media metrics such as reach, impressions, and number of views.

Composite scores for the Willingness to Intervene Against Suicide subscales and the Internet Self-Efficacy Scale were calculated, and negatively worded items were reverse-coded. Friedman’s test was used to determine statistically significant change in median scores between timepoints. Median scores and nonparametric tests were used as the data violated assumptions of normality (Shapiro-Wilk test). If statistical significance was observed, post hoc analysis with Wilcoxon signed ranks test was conducted to determine whether the change occurred between T1 and T2, or T2 and T3. A conservative Bonferroni correction was calculated by dividing the significance level (0.05) by the number of tests (3), resulting in level of significance set at p<0.017. P-values greater than 0.017 were considered not significant.

The Perceived Safety Questionnaire was analysed using McNemar’s test comparing differences in proportions of responses across timepoints where the outcome was dichotomous. For categorical outcomes, Pearson chi-square test for univariate frequency distribution was used to determine change in proportions of responses across timepoints.

For measures included in the T1, T2 and T3 questionnaires, subgroup analyses by age and gender were also conducted using McNemar’s test, Pearson chi-square test and Mann Whitney U test. All analyses were conducted using SPSS v25 software package and Stata/IC Version 15.1.

#### Sample size

As this was as an exploratory feasibility study and novel in design, no power calculation was used to determine sample size [44]. However, one of the main measures used in this study was created with sample sizes ranging from 172–367 [41]. Therefore, we used this to guide the target sample size.

## Results

### Participants

**Fig 4:**
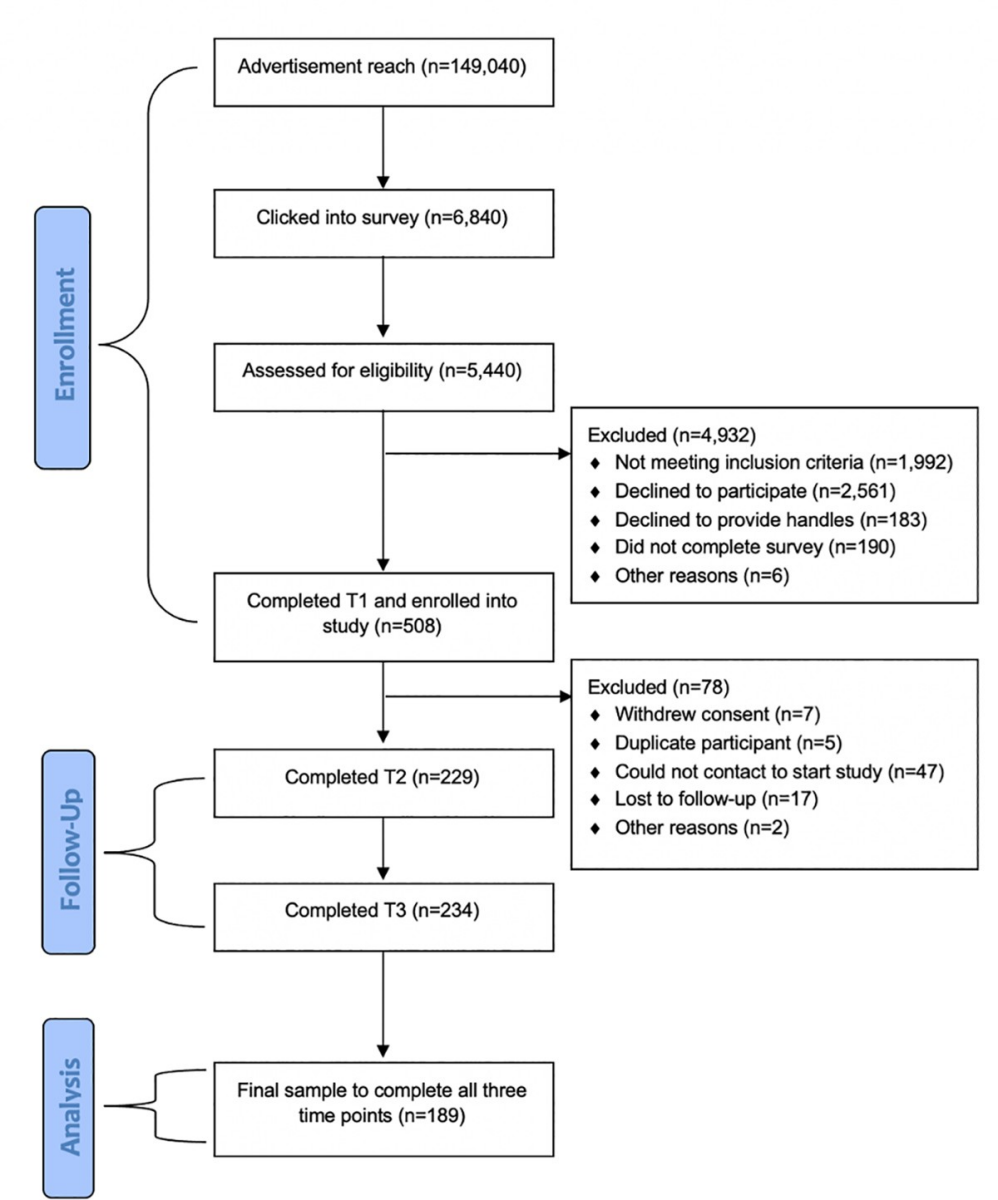
Participant flow diagram from enrolment, follow-up, and data analysis for the #chatsafe intervention.

A total of 6,840 young people responded to the advertisements and clicked into the survey. Of them 514 were eligible and completed the baseline questionnaire. Only participants who commenced the intervention and completed all three assessments were retained for analyses. This resulted in a final sample of 189 young people–see Fig 4. Participant demographics and social media usage are reported in Table 2.

**Table 2:** Demographic characteristics and baseline social media usage of the sample.

Characteristics	n (%)
**Age, M (SD)**	18.37 (2.63)
16–19 years	137 (72.49%)
20–25 years	52 (27.51%)
**Gender**	
Male	49 (25.93%)
Female	128 (67.72%)
Transgender	5 (2.65%)
Gender fluid	5 (2.65%)
Gender neutral	2 (1.05%)
**Sexual Orientation**	
Heterosexual	101 (53.44%)
Gay	15 (7.94%)
Bisexual	38 (20.12%)
Questioning	13 (6.88%)
Other	22 (11.64%)
**Nationality**	
Australian	161 (85.19%)
Other	28 (14.81%)
**Aboriginal and/or Torres Strait Islander**	
Yes	2 (1.05%)
No	187 (98.94%)
**Currently studying or training**	
Yes	154 (81.48%)
No	33 (17.46%)
**Currently employed**	
Yes	74 (39.15%)
No	115 (60.84%)
Time spent on social media^a^	
Less than 1 hour	3 (1.59%)
1–2 hours	39 (20.63%)
2–3 hours	67 (35.45%)
3–4 hours	54 (28.57%)
More than 5 hours	26 (13.76%)

^a^ Derived from Patterns of Social Media Use questionnaire.

Eligible participants indicated one or more of the following: they had used social media to talk about suicide (n = 96, 50.79%), they had viewed suicide-related content on social media (n = 169, 89.42%), they had wanted to talk about suicide on social media but did not feel equipped to do so (n = 72, 38.10%), and/or they managed, or were part of, a suicide discussion, bereavement, or memorial group online (n = 11, 5.82%).

Participants retained for analyses did not significantly differ from those not retained on any demographic variables.

### Key findings

#### Acceptability

*Weekly acceptability data*. The content themes for each week can be seen in Table 1 and reactions to the content can be seen in Table 3. As can be seen in Table 3, weekly acceptability responses decrease each week (from 84.66% during Week 1 to 59.79% at Week 11) followed by an increase in responses at Week 12 (67.72%).

**Table 3:** Weekly evaluations of the social media content shared within the #chatsafe intervention.

Week	Question 1^a^		Question 2^b^		Question 3^c^		Total responses (N)	Adherence^d^, %
	Positive, n (%)	Negative, n (%)	Positive, n (%)	Negative, n (%)	Positive, n (%)	Negative, n (%)		
1	149 (93.13)	1 (0.63)	99 (61.88)	38 (23.75)	129 (80.63)	6 (3.75)	160	84.66
2	**157 (96.91)**	2 (1.23)	127 (78.40)	20 (12.35)	143 (88.27)	6 (3.70)	162	85.71
3	114 (78.62)	13 (8.97)	89 (61.38)	32 (22.07)	97 (66.90)	9 (6.21)	145	76.72
4	108 (78.26)	11 (7.97)	79 (57.25)	36 (26.09)	102 (73.91)	6 (4.35)	138	73.02
5	108 (81.20)	12 (9.02)	93 (69.92)	27 (20.30)	90 (67.67)	**15 (11.28)**	133	70.37
6	96 (71.64)	**20 (14.93)**	84 (62.69)	30 (22.39)	91 (67.91)	14 (10.45)	134	70.90
7	123 (93.89)	5 (3.82)	100 (76.34)	18 (13.74)	**116 (88.55)**	4 (3.05)	131	69.31
8	97 (75.19)	17 (13.18)	78 (60.74)	**36 (27.91)**	96 (74.42)	10 (7.75)	129	68.25
9	121 (96.80)	3 (2.40)	**101 (80.80)**	16 (12.80)	110 (88.00)	11 (8.80)	125	66.14
10	102 (82.26)	9 (7.26)	80 (64.52)	26 (20.97)	96 (77.42)	5 (4.03)	124	65.61
11	105 (92.92)	3 (2.65)	85 (75.22)	12 (10.62)	86 (76.11)	7 (6.19)	113	59.79
12	112 (87.50)	5 (3.91)	91 (71.09)	25 (19.53)	110 (85.94)	2 (1.56)	128	67.72

**Note:** Positive sums were calculated by combining responses to ratings 4 or 5 (see Fig 3). Negative sums were calculated by combining responses to ratings 1 or 2 (see Fig 3). The number in the total column represents the total number of responses received that week. Boldface indicates the highest and lowest evaluations.

^a^ What did you think about the campaign content this week?

^b^ Would you share this week’s campaign content with your contacts on social media?

^c^ How did the campaign content you received today make you feel?

^d^ Adherence refers to the proportion of the total sample that engaged with each week’s content evaluations.

Overall, participants reported that their most preferred peice of content was Week 2 (an animation encouraging users to self-care; n = 157, 96.91) and that they were most likely to share Week 9’s campaign content (an animation containing practical tips on how to safely memorialize someone who had died by suicide; *n* = 101, 80.80%). Participants also reported that Week 7 content made them feel most positive (an animation encouraging users to pause and reflect before posting; n = 116, 88.55%).

Conversely, participants reported that their least preferred piece of content was from Week 6 (a video on self-care; *n* = 20, 14.93%), that they would be least likely to share the content from Week 8 (a video on self-care; *n* = 36, 27.91%), and that the Week 5 content (a text tile about safe posting on social media; *n* = 15, 11.28%) was associated with the most negative feelings.

Few associations were observed between individual-level characteristics and participant evaluations of the #chatsafe campaign content. An increase in age was associated with more negative feelings towards Week 1’s campaign content, with an odds ratio of 0.822 (95%CI 0.764, 0.995), *Wald’s* χ^2 (1) = 4.147, *p* = 0.042. An increase in age was also associated with more negative evaluations (OR: 0.861 (95%CI 0.748, 0.990), *Wald’s* χ^2 (1) = 4.431, *p* = 0.035) and more negative feelings towards Week 3’s campaign content (OR: 0.850 (95% CI 0.740, 0.975), *Wald’s* χ^2 (1) = 5.363,p = 0.021. An increase in age was associated with more positive evaluations of Week 7’s campaign content, with an odds ratio of 1.178 (95% CI 1.004, 1.382), *Wald’s* χ^2 (1) = 4.058, *p* = 0.044.

No gender differences were observed.

*Post-intervention evaluation data*. At the end of the intervention (T2), 80% of participants (*n* = 150) reported that they found the campaign content helpful for themselves; with 32% (*n* = 60) reporting that it was ‘moderately’ helpful, 38% (*n* = 72) reporting it to be ‘very’ helpful, and 10% (*n* = 18) reporting it to be ‘extremely’ helpful. Participants also reported that they thought the content would be either ‘very’ or ‘extremely’ helpful for others (44%, *n* = 83 and 29%, *n* = 55 respectively). Forty-four per cent (*n* = 83) reported that their confidence talking online about suicide was moderately improved as a result of the intervention, 30% (*n* = 57) reported it to be ‘highly’ improved, and 11% (*n* = 21) reported that the intervention ‘extremely’ improved their confidence.

Finally, participants were asked if the content had a negative effect on them, or if they believed it would have a negative effect on others. Seventy-eight percent of participants (*n* = 148) said that the campaign content did ‘not at all’ have a negative impact, 17% (*n* = 33) said that it ‘somewhat’ impacted them and 4% (*n* = 7) said it ‘moderately’ impacted them. When asked about others, 40% (*n* = 76) believed that the content would ‘not at all’ have a negative impact on others and a further 53% (*n* = 101) believed the content could have a ‘somewhat’ negative impact on others.

#### Safety

*Adverse events*. During the study period no SAEs and six AEs were recorded. Of the six AEs, three involved participants opting to withdraw from the study and three involved participants contacting the study team via direct message or a comment on the #chatsafe social media platforms, expressing their own (or someone else’s) experience of past or current suicidality. These participants were contacted by a member of the study team and they were provided helplines and/or the opportunity speak with the study psychologist.

In addition, out of a maximum potential of 5,160 weekly responses, 2,451 responses were recorded. Of these, only four responses included a withdrawal request (with no distress recorded at follow up) and 31 included a snooze request. At no point during the study was it deemed necessary to remove any content from the #chatsafe social media pages.

#### Feasibility

*Recruitment and adherence*. The response rate for study completion (defined as completing T1, T2 and T3) was 189/430 (43.95%). Despite a high attrition rate, this study was able to recruit a sufficient sample size to investigate the feasibility and acceptability of the #chatsafe intervention.

*Reach*. Throughout the 12-week #chatsafe campaign that ran parallel to this study, 1,430,789 individuals were reached through the #chatsafe social media platforms, both through organic sharing of the content and paid advertising [45]. The #chatsafe content was shown a total of 3,796,978 times on social media between October 2019 and January 2020. Snapchat and Instagram were the two best performing platforms, followed by Facebook, YouTube and Twitter. Each Instagram post received a mean of 67 likes and each animation/video was watched an average of 365 times. Videos on the #chatsafe YouTube page were viewed 151,023 times.

#### Willingness to intervene against suicide online

Table 4 presents the median scores for the Willingness to Intervene Against Suicide and Internet Self-Efficacy measures for the whole sample. Table 5 presents the sub-group analyses by gender and age. Only data for male and female participants were able to be analyzed, as the sample of participants with other gender identities was too small to allow for robust analyses (see Table 1).

**Table 4:** Ability and willingness to intervene against suicide online and internet self-efficacy for the entire sample.

	N	Median	IQR	Z statistic	P-value
**Ability to intervene ^a^**					
**T_1_**	189	74.00	65.00–81.00		
**T_2_**	189	81.00	73.00–87.00	-6.75	< .001
**T_3_**	189	81.00	74.00–88.00	-2.48	.013
**Intent to intervene ^a^**					
**T_1_**	189	84.00	76.00–90.00		
**T_2_**	189	88.00	80.00–95.00	-6.32	< .001
**T_3_**	189	88.00	79.00–95.00	-0.73	.464
**Reactive/generative self-efficacy ^b^**					
**T_1_**	189	30.00	26.00–34.50		
**T_2_**	189	32.00	27.00–36.00	-3.26	.001
**T_3_**	189	32.00	27.00–36.00	-0.31	.759
**Differentiation self-efficacy ^b^**					
**T1**	189	22.00	20.00–25.00		
**T2**	189	24.00	21.00–28.00	-3.36	.001
**T3**	189	24.00	20.00–26.00	-0.20	.844
**Organisation self-efficacy ^b^**					
**T1**	189	18.00	15.00–20.00		
**T2**	189	18.00	16.50–21.00	-2.82	.005
**T3**	189	18.00	17.00–21.00	-1.74	.081
**Communication self-efficacy ^b^**					
**T1**	189	12.00	10.00–13.00		
**T2**	189	12.00	10.00–13.00		
**T3**	189	12.00	10.00–13.00		
**Search self-efficacy ^b^**					
**T1**	189	13.00	11.00–13.00		
**T2**	189	12.00	12.00–14.00		
**T3**	189	13.00	11.00–14.00		

^a^ Derived from the Willingness to Intervene Against Suicide questionnaire.

^b^ Derived from the Internet Self-efficacy Scale.

**Table 5:** Sub-group analyses for ability and willingness to intervene against suicide online.

		N	Median	IQR	Z statistic	P-value
**Ability to intervene ^a^**						
**Male**	T_1_	49	74.00	66.50–82.50		
	T_2_	49	80.00	74.50–87.00	-3.96	< .001
	T_3_	49	82.00	72.00–86.50	-0.33	.739
**Female**	T_1_	128	74.50	65.00–81.00		
	T_2_	128	81.00	72.25–87.00	-5.42	< .001
	T_3_	128	81.00	75.00–88.00	-2.48	.013
**<20-years**	T_1_	137	74.00	64.00–81.00		
	T_2_	137	80.00	73.00–87.00	-6.07	< .001
	T_3_	137	80.00	73.50–87.00	-1.78	.075
**≥20-years**	T_1_	52	75.00	67.00–85.00		
	T_2_	52	82.00	72.75–87.75	-2.96	.003
	T_3_	52	81.00	75.00–89.75	-1.94	.053
**Intent to intervene ^a^**						
**Male**	T_1_	49	68.00	62.00–74.50		
	T_2_	49	72.00	68.00–77.50	-4.37	< .001
	T_3_	49	71.00	64.50–79.00	-0.99	.322
**Female**	T_1_	128	70.00	65.00–76.75		
	T_2_	128	74.00	68.25–82.00	-4.61	< .001
	T_3_	128	74.00	69.00–80.75	-0.51	.613
**<20-years**	T_1_	137	84.00	76.00–89.00		
	T_2_	137	87.00	80.00–95.00	-5.41	< .001
	T_3_	137	87.00	79.00–95.00	-0.61	.541
**≥20-years**	T_1_	52	84.00	74.25–93.75		
	T_2_	52	89.00	81.00–96.50	-3.31	.001
	T_3_	52	88.00	78.75–97.50	-0.682	.495

^a^ Derived from the Willingness to Intervene Against Suicide questionnaire.

It should be noted that participants who completed all three assessments demonstrated a greater increase in their intent to intervene against suicide compared to participants who only completed the first two assessments (*U* = 4554.00, *p* = 0.036). No other differences were observed in any of the outcome variables.

*Ability to intervene*. There was a change in participants’ perceived ability to respond to someone who may be suicidal from T1 to T3, *χ*^*2*^ (2) = 75.57, *p<* .001. An increase in ability was observed from T1 to T2 by 9.46% (*Z* = -6.744, *p<* .001). No changes were observed from T2 to T3. Increases in ability to intervene from T1 to T2 were observed in males by 7.50% (Z = -3.96, p<0.001) and females by 9.46% (Z = -5.41, p<0.001), and in both the < 20-years and ≥ 20-years age groups by 7.50% and 9.33%, respectively (<20 years: Z = -6.07, p<0.001 and ≥20 years: Z = -2.96, p = 0.003). However, the ≥ 20-years age group reported a greater ability to intervene at all timepoints compared to the < 20-years age group.

*Intent to intervene*. There was a change in participants’ intent to respond to someone who may be suicidal from T1 to T3, *χ*^*2*^ (2) = 36.36, *p* < .001. There was an increase in participants’ intent to intervene from T1 to T2 by 4.76% (*Z* = -6.324, *p* < .001) and no change from T2 to T3. Increases in intent to intervene were observed in males by 5.88% (Z = -4.37, p<0.001) and females by 5.71% (Z = -4.61, p<0.001), however female participants indicated a greater intent to intervene across all three timepoints. Increases were also observed in both the < 20-years age group by 3.57% (Z = -5.41, p<0.001) and ≥ 20-years age group by 5.95% (Z = -3.31, p = 0.001).

#### Internet self-efficacy, confidence and safety when communicating about suicide online

*Internet self-efficacy*. A change was observed in three subscales of this measure: reactive/generative, differentiation, and organisation self-efficacy across the timepoints (reactive/generative: χ^2^ (2) = 16.40, *p* < .001, differentiation: χ^2^ (2) = 17.91, *p* < .001, and organisation: χ^2^ (2) = 14.38, p = .001). Increases were observed in females and the <20 years age group for reactive/generative, differentiation, and organisation self-efficacy between T1 and T2. There was also an increase for males in reactive/generative self-efficacy from T1 to T2. No changes were observed from T2 to T3. See S1 Table for subgroup analyses for each domain.

*Perceived confidence and safety*. At all three timepoints, the majority of participants responded that they ‘rarely’ or ‘never’ created, shared or liked posts involving suicide content. Change was observed in the distribution of responses T1 to T2, χ^2^ (4) = 15.49, *p* = .004, and from T2 to T3, χ^2^ (4) = 17.42, *p* = .002. The proportion of participants who indicated that in the past month they ‘sometimes’ created, liked or shared a post involving suicide content decreased between timepoints, and the proportion of respondents who indicated that in the past month they ‘never’ created, liked or shared a post involving suicide content increased between timepoints. No age or gender differences were observed.

Of those who indicated that they did create a post involving suicide-related content at each of the timepoints, the proportion of participants who monitored their post for unsafe content increased from T1 to T2, χ^2^ (1) = 58.84, *p* < .001, with no change from T2 to T3. The majority of participants in both the < 20-years and ≥20-years age groups indicated that they monitored their post across all time points. There was an increase in monitoring in both males and females from T1 to T3. When asked how they responded to unsafe content, the most common actions were to delete or hide the post and/or to contact the person who made the post.

Table 6 presents the type of online suicide-related social media content seen by participants on their social media feeds during the course of the study. The most common form of suicide-related content that participants reported seeing at T1 were statements that appeared to ‘deliberately seek to trigger difficult or distressing emotions in other people’. The majority of participants reported viewing at least one form of suicide-related content during the course of the study, most commonly ‘graphic descriptions of suicide’.

**Table 6:** Forms of suicide-related social media content seen by participants across timepoints.

**Form of suicide-related content**	T1		T2		T3	
	**N**	%	N	%	N	%
**Graphic descriptions of suicide**	38	20	105	56	98	52
**Graphic images of suicide**	23	12	17	9	14	7
**Means or methods of suicide**	66	35	51	27	42	22
**Plans of suicide**	44	23	33	17	25	13
**Statements that encourage people to take their own life**	45	24	32	17	31	16
**Statements that appear to deliberately seek to trigger difficult or distressing emotions in other people**	67	35	61	32	50	26
**Statements that include suicide pacts or suicide partners**	14	7	6	3	11	6
**Statements that place blame or make others feel responsible for another person’s safety**	51	27	39	21	31	16
**Statements that provide vulnerable people information about how to end their life**	23	12	17	9	16	8
**Suicide notes or goodbye notes**	44	23	37	20	36	19

As shown in Table 7, despite participants reporting that they frequently viewed online content related to suicidal behaviour, the majority reported that the content did not make them believe that the creator of that post was at risk of suicide. Those who had seen a post that made them concerned were asked how they responded. There was a difference in responses from T1 to T2, χ^2^ (6) = 18.88, *p* = .004, and from T2 to T3, χ^2^ (6) = 20.29, *p* = .002. Most apparent was the increase over time in the proportion of participants who responded directly to the person. Subgroup analyses indicated that for both age groups they would most likely reach out to the person or report the post to the social media platform (see S2 Table). At all three timepoints, female participants most commonly responded by directly contacting the creator of the post. At T1 and T2 male participants were more likely to report the post to the social media platform, but at T3, were more likely to contact the person directly. Participants were more likely to report that they felt capable of responding to someone who was at risk immediately post-intervention (*n* = 107, 71%), compared to baseline (*n* = 102, 63%).

**Table 7:** Select questions and responses from the Perceived Safety questionnaire across timepoints.

**Question and response for total sample**		T1 N (%)	T2 N (%)	T3 N (%)
**How often did participants create, share or like posts involving suicide content?**				
	Often	16 (8.47%)	5 (2.65%)	8 (4.23%)
	Sometimes	63 (33.33%)	56 (29.63%)	47 (24.87%)
	Rarely	70 (37.04%)	76 (40.21%)	59 (31.22%)
	Never	37 (19.58%)	51 (26.98%)	74 (39.15%)
	Prefer not to answer	3 (1.59%)	1 (0.53%)	1 (0.53%)
**Did participants monitor their post for unsafe content?**				
	Yes	73 (48.99%)	112 (81.75%)	89 (78.07%)
	No	76 (51.01%)	25 (18.25%)	25 (21.93%)
**Did participants see a post that made them think the creator of the post might be at risk of suicide?**				
	Often	10 (5.29%)	6 (3.17%)	5 (2.65%)
	Sometimes	77 (40.74%)	49 (25.93%	45 (23.81%)
	Rarely	76 (40.21%)	90 (47.62%)	85 (44.97%)
	Never	24 (12.70%)	41 (21.69%)	50 (26.46%)
	Prefer not to answer	2 (1.06%)	3 (1.59%)	4 (2.12%)
**How did participants respond to unsafe content on their post?**				
	Sought professional advice	2 (1.23%)	2 (1.38%)	1 (0.74%)
	Responded to the person directly	48 (29.45%)	52 (35.86%)	46 (34.07%)
	Informed a trusted adult or friend	6 (3.68%)	12 (8.28%)	4 (2.96%)
	Contacted the relevant platform safety centre	5 (3.07%)	6 (4.14%)	14 (10.37%)
	Did not respond	60 (36.81%)	33 (22.76%)	38 (28.15%)
	Other	1 (0.61%)	1 (0.69%)	1 (0.74%)
	A combination of responses	41 (25.15%)	39 (26.90%)	31 (22.96%)

## Discussion

This was the first study to examine a suicide prevention campaign specifically designed for young people and delivered entirely through social media. The study found the #chatsafe campaign to be acceptable, safe and feasible. Following the campaign, participants reported being more willing to intervene against suicide, and reported greater self-efficacy, confidence and perceived safety when communicating on social media about suicide. The #chatsafe intervention also appeared to improve aspects of online behaviour, with participants reporting being: less likely to share suicide-related content; more likely to monitor their posts for harmful content; and being more likely to contact someone directly if they believed they were at risk, following the intervention. The trends observed in this study not only improved immediately following the delivery of the #chatsafe intervention but were maintained at a four-week follow up. This suggests that the impact of the #chatsafe intervention has the potential to be maintained over time.

Findings from this study also support that young people are viewing online suicide-related content at an increasing rate [22], including graphic depictions of self-harm, which is widely considered to be potentially harmful [29]. Although survey items in this study mostly referred to suicide-related content created by participants’ peers or online networks, suicide-related content can appear on young people’s news feeds without prior warning. A recent example was the live streaming of a suicide on the social media platform, TikTok, which was viewable by their estimated 328 million users under the age of 24 [46, 47]. Prior studies report that exposure to unsafe and poorly moderated suicide-related content were associated with an increase in young people experiencing suicidal ideation and suicide attempts [48]. This speaks to the need for young people to feel equipped in knowing how to manage the content they encounter and the findings from this study suggest that the #chatsafe intervention can play a useful part in this process.

### Implications

Until now, despite significant debate about the relationship between social media and young people’s mental health [49–54], there has been a paucity of research examining the potential effectiveness of social media interventions in youth suicide prevention, and much of the existing evidence pertaining to safe communication about suicide has arisen from studies involving mainstream media [53–55]. However, it can be argued that young people use social media to communicate about suicide in fundamentally different ways when compared to mainstream media. Critically, young people tend not to use social media to consume news; rather, their online behaviours are more dynamic–to build a sense of community by sharing their feelings with others who have had similar experiences, to seek help and to provide help to others, and to express grief for people who have died by suicide [16, 17, 55]. As a result, the knowledge gained from previous research examining mainstream media may not apply here. Our findings suggest that rather than being harmful, delivering suicide prevention content via social media can be acceptable, safe and feasible to do. Moreover, it may be associated with notable benefits.

After receiving the 12-week #chatsafe intervention, participants in this study reported an increase in: their willingness to intervene against suicide online; aspects of their internet self-efficacy; and perceived confidence and safety when communicating online about suicide. Although these improvements were reported for all participants, females and participants aged over 20 years recorded the greatest increases, suggesting that slightly different content may resonate better with younger people and males. Moreover, females and younger participants’ internet self-efficacy saw the greatest increases, particularly with their ability to organize information online (e.g., retain control of the information they want or do not want to see), their ability to find and share information, and their perceived ability to create appropriate content to share with others. Again, this suggests that different types of content resonates differently across the population.

Most of the content was well received by participants and the most preferred pieces of content were animation videos that encouraged users to practice self-care online, provided them with practical tips on how to talk safely about someone who has died by suicide, and encouraging them to pause and reflect before posting suicide-related content on social media. These positive evaluations of this type of content support previous public health campaigns that suggest content that evokes emotional responses and assist learning resonate most strongly with young people. Further, considerable attention is paid to the potential negative impact of media reporting following the suicide of both a public figure or member of the community, with research suggesting that exposure to sensationalist or graphic content can cause harm and potentially contribute to the development or maintenance of suicide clusters [56, 57]. Thus, access to information on safe ways to communicate about someone who has died by suicide might go some way to mitigating the risk of future suicide clusters.

In contrast, and somewhat surprisingly, the content that participants evaluated least favorably, and were least likely to share, related to self-care. As described above, every second week, self-care content was sent to the participants, as opposed to content specific to suicide. The reasons for this were to reduce the risk of over-exposure to suicide-related content and any associated distress or risk. However, the findings suggest that young people were more likely to share the more ‘active’ content that included practical tips and advice compared to the more benign self-care content. This reiterates the findings from our earlier study that reported on the development of the #chatsafe social media content [30], in which young people specifically requested that the #chatsafe campaign was not simply “another awareness campaign” but actually provided them with tangible skills to help themselves and each other. It also supports earlier work exploring the effectiveness of digital health interventions, which reported that participants favoured content that taught them something they did not already know [58, 59]. While there have been a number of suicide awareness campaigns previously, they appear to have limited capacity to shift behaviour [35, 60], therefore a campaign, such as the one reported here, may stand to have greater utility for young people.

The emphasis on self-care content was part of the safety strategy associated with this study. There are unique safety and ethical challenges that exist when including young people in suicide research, that may be amplified when interventions are delivered online [12]. However, this study found that the #chatsafe intervention was not only well received by young people, it was also safe, and there are likely a number of reasons for this. In addition to sharing self-care and general wellbeing content throughout the intervention, a robust safety protocol was established whereby any time an AE was recorded, the research team met with the safety committee. This was to ensure that the safety protocol designed for this study was sufficient and to establish that the AEs were not attributable to the #chatsafe intervention. Second, the campaign was delivered universally, and as such did not set out to target those at elevated risk. That being said, although participants largely felt that the campaign did not have a negative impact on themselves, it is important to recognize that there were some participants who believed that the content may have a negative impact on others. This likely supports the fact that young people are aware that exposure to suicide-related content online can cause distress and remains a sensitive topic [30]. Together, this adds to a growing body of literature that suggests that it can be safe to involve young people in suicide prevention research, including research testing online interventions [61]. It also suggests that suicide prevention social media campaigns can be both safe and potentially effective as a suicide prevention strategy in the future.

A key benefit of social media is its capacity to reach large numbers of people quickly. The metrics relating to the campaign that ran in parallel to this study, suggest that approximately 1.5 million young people were exposed to the #chatsafe content in a three-month period. Following the current study, we received funding to adapt the guidelines and social media content for an additional 10 regions around the world, which reached a further 1 million individuals over a six-week period [45]. This has clear implications for the widescale delivery of information relating to suicide prevention both in Australia and worldwide. It also raises questions as to whether campaigns such as #chatsafe could in fact be used as a way of directing young people to clinical services or as part of a real-time response following the suicide of a young person, or for providing health-related information, particularly in low resource areas.

This could be critical as it is known that many young people at risk of suicide do not seek professional help, and among those that do, many get turned away without receiving adequate care [62]. Whilst the Australian government, at both state and federal level, is attempting to address this by providing additional resources for mental health services [63, 64], there is still an urgent need for community-based interventions that can reach large numbers of young people quickly and provide them with much-needed skills and information.

### Strengths and limitations

A key strength of this study was that the intervention was entirely co-designed with young people [30]. Although the importance of co-design is becoming increasingly recognized [65, 66], it is rare in youth suicide prevention [16, 67]. In this study young people were active partners and this likely contributed to the acceptability, safety and impact of the #chatsafe intervention.

An additional strength relates to feasibility; the study was able to recruit an appropriate sample size and whilst a high attrition rate was recorded, this is not uncommon in psycho-educational online interventions [68]. Also, while signing up to a study such as this is quite simple, the burden associated with weekly or time-based responses often results in higher attrition rates particularly with younger participants [69, 70] and in longer studies [71]. Despite this, a large sample size was initially recruited into the study and a sufficient sample size was retained across the duration of the 20-week period. It would be beneficial for future studies to examine why participants do drop out in order to be certain that it does relate to burden and not safety or acceptability.

There are however a number of limitations. First, relates to study design. This was an exploratory, and not a controlled, study, and as such it is not certain that the changes observed were the result of the #chatsafe intervention. That said the findings from this feasibility study will inform a larger, controlled study due to commence in 2021.

Second, the data collected were entirely self-reported. This poses issues relating to participant recall and subjectivity of the data. Similar to all studies investigating social media behaviour, more objective measures of social media usage are required as inaccurate retrospective self-reports of behaviour are common in internet-based research [72]. However, despite the T1, T2 and T3 surveys relying on retrospective reporting, the weekly assessments included in this study had methodological strengths. In using a short momentary-assessment each week, participant recall bias was minimised and this also allowed the research team to collect reactions to the intervention content in real-time. This was particularly useful when attempting to monitor levels of distress, risk, and engagement of study participants in a novel intervention. To this end, future studies should attempt to collect more objective measures of social media behaviour and minimise the time frame between survey responses and the behaviour being investigated [69, 73].

Third, the sample recruited to this study was not fully representative of the Australian population, and self-selection into this study may have biased the findings. In particular, there was a higher proportion of females and a higher proportion of non-heterosexual young people than in the general community. Although the national campaign managed to reach similar numbers of males and females, there was an underrepresentation of young males in the study sample. This is not unusual in suicide prevention research [16] but does warrant attention in future studies, particularly given the over-representation of males in the suicide statistics [1]. Also, despite having partnered closely with young people from culturally and linguistically diverse, and Aboriginal and Torres Strait Islander, backgrounds in the co-design process [30], both of these groups were under-represented in the study. In determining eligibility for this study, over half of the sample had previously used social media to talk about suicide and just over a third of the sample indicated that they had wanted to talk about suicide on social media but did not feel equipped to do so. Collecting data from young people who already had experience in using social media to communicate about suicide may have produced a sample of young people who self-selected into the study due to an interest in the subject matter. As a result, these findings may not apply to all young people.

Fourth, although the data indicate an increase in perceived ability, intent, confidence and safety when communicating online about suicide, it is unknown if this impacted on actual behaviour. The next phase of this study will address these limitations by objectively coding social media data collected from participants. This will allow a direct comparison between the nature of suicide-related communication prior to, during, and after exposure to the #chatsafe campaign content.

There are also difficulties in measuring the precise level of engagement by study participants as social media metrics could not differentiate between study participants and the general public and there was no way of knowing how much or how little the participants interacted with the #chatsafe content. While it was possible to see that the direct messages sent to the participants’ social media platforms were ‘opened’, ‘read’, or ‘received’, it was not possible to measure the amount of time that participants spent viewing the content, or whether they clicked through to the #chatsafe website for more information. Indeed, this reflects the amorphous nature of social media platforms. However, the content did reach a large number of people in a short period of time and there was no indication that the content was harmful to anyone who came across it. Finally, the adoption of an emoji scale, while appropriate and familiar to a younger demographic, did make it difficult to interpret specific mood states [74].

Despite its limitations, this study has demonstrated that it is feasible, safe and acceptable to use social media for the purpose of suicide prevention. It also provided promising evidence for the potential impact of social media campaigns on increasing young people’s digital safety when it comes to suicide prevention. Although there are challenges associated with measuring real-world interventions in real-time and across uncontrolled settings [12, 75, 76], this study has provided important data which will inform a larger-scale, and more rigorous study.

## Conclusions

Overall, findings from this study present a promising picture for the acceptability and impact of a universal suicide prevention campaign delivered through social media. Until now, little was known about the potential benefits of a social media campaign for suicide prevention. This study has demonstrated that it is safe, acceptable and feasible to share youth suicide prevention information via social media, and its findings also indicate that the #chatsafe intervention may have increased young people’s perceived capacity to intervene against suicide online, internet self-efficacy and perceived safety. The next steps will be to examine the impact of the #chastafe intervention on actual social media behaviour using a controlled study design. In the meantime, however, it would appear that the use of social media to educate and equip young people with suicide prevention information appears to be safe and effective.

## Supporting information

S1 Table Internet self-efficacy subgroup analyses by age and gender. (DOCX)

S2 Table Select questions from the Perceived Safety Questionnaire subgroup analyses by age and gender. (DOCX)

S1 Data (XLS)

S2 Data (XLSX)
