# Supplementary material for: Can a social media intervention improve online communication about suicide? A feasibility study examining the acceptability and potential impact of the #chatsafe campaign
Source: PLoS One. 2021 Jun 15;16(6):e0253278. doi: 10.1371/journal.pone.0253278 (PMC8205132; doi:10.1371/journal.pone.0253278)
Supplement: S1 Table — (DOCX) [file pone.0253278.s001.docx]

**S1 Table**

Internet self-efficacy by age group and gender

|  |  | N | Median | IQR | Z statistic | P-value |
| --- | --- | --- | --- | --- | --- | --- |
| Reactive/generative self-efficacy | |  |  |  |  |  |
| Male | T_1_ | 49 | 30.00 | 24.00-33.00 |  |  |
|  | T_2_ | 49 | 31.00 | 25.00-36.00 | -2.54 | .011 |
|  | T_3_ | 49 | 30.00 | 25.00-34.50 | -0.95 | .330 |
| Female | T_1_ | 128 | 31.00 | 26.00-35.00 |  |  |
|  | T_2_ | 128 | 32.00 | 28.00-36.00 | -2.52 | .012 |
|  | T_3_ | 128 | 33.00 | 28.00-37.00 | -0.866 | .387 |
| <20-years | T_1_ | 137 | 30.00 | 26.00-34.00 |  |  |
|  | T_2_ | 137 | 32.00 | 27.00-36.00 | -2.60 | .009 |
|  | T_3_ | 137 | 33.00 | 27.50-36.50 | -0.890 | .373 |
| ≥20-years | T_1_ | 52 | 30.50 | 25.00-35.75 |  |  |
|  | T_2_ | 52 | 32.00 | 28.00-36.00 |  |  |
|  | T_3_ | 52 | 31.00 | 27.00-35.75 |  |  |
| Differentiation self-efficacy | |  |  |  |  |  |
| Male | T_1_ | 49 | 23.00 | 20.00-26.00 |  |  |
|  | T_2_ | 49 | 23.00 | 21.00-28.00 |  |  |
|  | T_3_ | 49 | 24.00 | 21.00-26.50 |  |  |
| Female | T_1_ | 128 | 22.00 | 20.00-25.00 |  |  |
|  | T_2_ | 128 | 24.00 | 20.25-27.00 | -3.03 | .002 |
|  | T_3_ | 128 | 24.00 | 20.00-26.00 | -0.026 | .979 |
| <20-years | T_1_ | 137 | 22.00 | 19.00-24.00 |  |  |
|  | T_2_ | 137 | 23.00 | 20.00-26.00 | -3.16 | .002 |
|  | T_3_ | 137 | 23.00 | 20.00-26.00 | -0.432 | .666 |
| ≥20-years | T_1_ | 52 | 23.50 | 22.00-27.00 |  |  |
|  | T_2_ | 52 | 24.00 | 22.00-28.00 |  |  |
|  | T_3_ | 52 | 25.50 | 21.25-28.00 |  |  |
| Organisation self-efficacy | |  |  |  |  |  |
| Male | T_1_ | 49 | 18.00 | 15.50-20.00 |  |  |
|  | T_2_ | 49 | 18.00 | 16.50-20.00 |  |  |
|  | T_3_ | 49 | 18.00 | 17.00-20.50 |  |  |
| Female | T_1_ | 128 | 18.00 | 15.00-20.00 |  |  |
|  | T_2_ | 128 | 18.00 | 16.25-21.00 | -3.18 | .001 |
|  | T_3_ | 128 | 18.00 | 17.00-21.00 | -1.74 | .083 |
| <20-years | T_1_ | 137 | 18.00 | 15.00-20.00 |  |  |
|  | T_2_ | 137 | 18.00 | 16.00-20.00 | -2.32 | .020 |
|  | T_3_ | 137 | 18.00 | 17.00-21.00 | -2.41 | .016 |
| ≥20-years | T_1_ | 52 | 18.00 | 17.00-20.75 |  |  |
|  | T_2_ | 52 | 19.00 | 18.00-21.00 |  |  |
|  | T_3_ | 52 | 18.50 | 17.00-21.00 |  |  |
| Communication self-efficacy | |  |  |  |  |  |
| Male | T_1_ | 49 | 11.00 | 10.00-13.00 |  |  |
|  | T_2_ | 49 | 12.00 | 10.00-13.50 |  |  |
|  | T_3_ | 49 | 11.00 | 10.00-13.00 |  |  |
| Female | T_1_ | 128 | 12.00 | 10.00-13.00 |  |  |
|  | T_2_ | 128 | 12.00 | 11.00-13.00 |  |  |
|  | T_3_ | 128 | 12.00 | 11.00-14.00 |  |  |
| <20-years | T_1_ | 137 | 12.00 | 10.00-13.00 |  |  |
|  | T_2_ | 137 | 12.00 | 10.00-13.00 |  |  |
|  | T_3_ | 137 | 12.00 | 11.00-14.00 |  |  |
| ≥20-years | T_1_ | 52 | 11.00 | 8.00-13.00 |  |  |
|  | T_2_ | 52 | 12.00 | 10.25-13.00 |  |  |
|  | T_3_ | 52 | 11.50 | 10.00-13.00 |  |  |
| Search self-efficacy | |  |  |  |  |  |
| Male | T_1_ | 49 | 13.00 | 11.00-14.00 |  |  |
|  | T_2_ | 49 | 12.00 | 12.00-14.00 |  |  |
|  | T_3_ | 49 | 13.00 | 11.00-14.00 |  |  |
| Female | T_1_ | 128 | 13.00 | 11.00-13.00 |  |  |
|  | T_2_ | 128 | 13.00 | 12.00-14.00 |  |  |
|  | T_3_ | 138 | 13.00 | 12.00-14.00 |  |  |
| <20-years | T_1_ | 137 | 12.00 | 11.00-13.00 |  |  |
|  | T_2_ | 137 | 12.00 | 11.00-14.00 |  |  |
|  | T_3_ | 137 | 12.00 | 11.00-14.00 |  |  |
| ≥20-years | T_1_ | 52 | 13.00 | 12.00-14.00 |  |  |
|  | T_2_ | 52 | 13.00 | 12.00-14.00 |  |  |
|  | T_3_ | 52 | 13.00 | 12.00-14.00 |  |  |
