# Supplementary material for: Can a social media intervention improve online communication about suicide? A feasibility study examining the acceptability and potential impact of the #chatsafe campaign
Source: PLoS One. 2021 Jun 15;16(6):e0253278. doi: 10.1371/journal.pone.0253278 (PMC8205132; doi:10.1371/journal.pone.0253278)
Supplement: S2 Table — (DOCX) [file pone.0253278.s002.docx]

**S2 Table**

Select questions and responses from the Perceived Safety questionnaire across timepoints, by age group and gender

|  | | T1  N (%) | T2  N (%) | T3  N (%) |
| --- | --- | --- | --- | --- |
| How often did participants create, share or like posts in | |  |  |  |
| Males |  |  |  |  |
|  | Often | 3 (6.12%) | 2 (4.08%) | 2 (4.08%) |
|  | Sometimes | 13 (26.53%) | 12 (24.49%) | 9 (18.37%) |
|  | Rarely | 22 (44.90%) | 18 (36.73%) | 17 (34.69%) |
|  | Never | 10 (20.41%) | 17 (34.69%) | 21 (42.86%) |
|  | Prefer not to answer | 1 (2.04%) | 0 (0.00%) | 0 (0.00%) |
| Females |  |  |  |  |
|  | Often | 13 (9.42%) | 3 (2.34%) | 6 (4.69%) |
|  | Sometimes | 41 (29.71%) | 42 (32.81%) | 34 (26.56%) |
|  | Rarely | 46 (33.33%) | 52 (40.63%) | 37 (28.91%) |
|  | Never | 27 (19.57%) | 30 (23.44%) | 50 (39.06%) |
|  | Prefer not to answer | 1 (0.72%) | 1 (0.78%) | 1 (0.78%) |
| <20-years |  |  |  |  |
|  | Often | 9 (6.57%) | 5 (3.65%) | 6 (4.38%) |
|  | Sometimes | 51 (37.23%) | 46 (33.58%) | 41 (29.93%) |
|  | Rarely | 48 (35.04%) | 54 (39.42%) | 45 (32.85%) |
|  | Never | 26 (18.98%) | 32 (23.36%) | 1 (32.12%) |
|  | Prefer not to answer | 3 (2.19%) | 0 (0.00%) | 1 (0.73%) |
| ≥20-years |  |  |  |  |
|  | Often | 7 (13.46%) | 0 (0.00%) | 2 (3.85%) |
|  | Sometimes | 12 (23.08%) | 10 (19.23%) | 6 (11.54%) |
|  | Rarely | 22 (42.31%) | 22 (42.31%) | 14 (26.92%) |
|  | Never | 11 (21.15%) | 19 (36.54%) | 30 (57.69%) |
|  | Prefer not to answer | 0 (0.00%) | 1 (1.92%) | 0 (0.00%) |
| Did participants monitor their post for unsafe content? | |  |  |  |
| Males |  |  |  |  |
|  | Yes | 15 (39.47%) | 26 (81.25%) | 20 (71.43%) |
|  | No | 23 (60.53%) | 6 (18.75%) | 8 (28.57%) |
| Females |  |  |  |  |
|  | Yes | 51 (51.00%) | 78 (80.41%) | 62 (80.52%) |
|  | No | 49 (49.00%) | 19 (19.59%) | 15 (19.48%) |
| <20-years |  |  |  |  |
|  | Yes | 58 (53.70%) | 84 (80.00%) | 70 (76.09%) |
|  | No | 50 (46.30%) | 21 (20.00%) | 22 (23.91%) |
| ≥20-years |  |  |  |  |
|  | Yes | 15 (36.59%) | 28 (87.50%) | 19 (86.36%) |
|  | No | 26 (63.41%) | 4 (12.50%) | 3 (13.64%) |
| Did participants see a post that made them think the creator of the post might be at risk of suicide? | |  |  |  |
| Males |  |  |  |  |
|  | Often | 1 (2.04%) | 0 (0.00%) | 0 (0.00%) |
|  | Sometimes | 14 (28.57%) | 10 (20.41%) | 11 (22.45%) |
|  | Rarely | 27 (55.10%) | 24 (48.98%) | 26 (53.06%) |
|  | Never | 6 (12.24%) | 14 (28.57%) | 12 (24.49%) |
|  | Prefer not to answer | 1 (2.04%) | 1 (2.04%) | 0 (0.00%) |
| Females |  |  |  |  |
|  | Often | 7 (5.47%) | 6 (4.69%) | 5 (3.91%) |
|  | Sometimes | 57 (44.53%) | 34 (26.56%) | 28 (21.88%) |
|  | Rarely | 46 (35.94%) | 61 (47.66%) | 55 (42.97%) |
|  | Never | 17 (13.28%) | 25 (19.53%) | 36 (28.13%) |
|  | Prefer not to answer | 1 (0.78%) | 2 (1.56%) | 4 (3.13%) |
| <20-years |  |  |  |  |
|  | Often | 9 (6.57%) | 5 (3.65%) | 4 (2.92%) |
|  | Sometimes | 57 (41.61%) | 39 (28.47%) | 29 (21.17%) |
|  | Rarely | 53 (38.69%) | 66 (48.18%) | 68 (49.64%) |
|  | Never | (16 (11.68%) | 24 (17.52%) | 32 (23.36%) |
|  | Prefer not to answer | 2 (1.46%) | 3 (2.19%) | 4 (2.92%) |
| ≥20-years |  |  |  |  |
|  | Often | 1 (1.92%) | 1 (1.92%) | 1 (1.92%) |
|  | Sometimes | 20 (38.46%) | 10 (19.23%) | 16 (30.77%) |
|  | Rarely | 23 (44.23%) | 24 (46.15%) | 17 (32.69%) |
|  | Never | 8 (15.38%) | 17 (32.69%) | 18 (34.62%) |
|  | Prefer not to answer | 0 (0.00%) | 0 (0.00%) | 0 (0.00%) |
| How did participants respond to unsafe content on their post? | |  |  |  |
| Males |  |  |  |  |
|  | Sought professional advice | 1 (2.08%) | 2 (4.44%) | 1 (2.27%) |
|  | Responded to the person directly | 15 (31.25%) | 15 (33.33%) | 14 (31.82%) |
|  | Informed a trusted adult or friend | 2 (4.17%) | 11 (24.44%) | 3 (6.82%) |
|  | Contacted the relevant platform safety centre | 3 (6.25%) | 6 (13.33%) | 8 (18.18%) |
|  | Did not respond | 25 (52.08%) | 10 (22.22%) | 16 (36.26%) |
|  | Other | 2 (4.17%) | 1 (2.22%) | 2 (4.55%) |
| Females |  |  |  |  |
|  | Sought professional advice | 7 (4.61%) | 8 (5.80%) | 7 (6.19%) |
|  | Responded to the person directly | 60 (39.47%) | 62 (44.93%) | 53 (46.90%) |
|  | Informed a trusted adult or friend | 18 (11.84%) | 24 (17.39%) | 16 (14.16%) |
|  | Contacted the relevant platform safety centre | 9 (5.92%) | 14 (10.14%) | 13 (11.50%) |
|  | Did not respond | 52 (34.21%) | 29 (21.01%) | 23 (20.35%) |
|  | Other | 6 (3.95%) | 1 (0.72%) | 1 (0.88%) |
| <20-years |  |  |  |  |
|  | Sought professional advice | 16 (13.22%) | 18 (14.63%) | 18 (14.75%) |
|  | Responded to the person directly | 10 (8.26%) | 20 (16.26%) | 15 (12.30%) |
|  | Informed a trusted adult or friend | 32 (26.45%) | 31 (25.20%) | 29 (23.77%) |
|  | Contacted the relevant platform safety centre | 6 (4.96%) | 9 (7.32%) | 8 (6.56%) |
|  | Did not respond | 28 (23.14%) | 33 (26.83% | 31 (25.41%) |
|  | Other | 29 (23.97%) | 12 (9.76%) | 21 (17.21%) |
| ≥20-years |  |  |  |  |
|  | Sought professional advice | 2 (6.06%) | 4 (14.81%) | 4 (10.53%) |
|  | Responded to the person directly | 3 (9.09%) | 3 (11.11%) | 4 (10.53%) |
|  | Informed to a trusted adult or friend | 9 (27.27%) | 1 (3.70%) | 15 (39.47%) |
|  | Contacted the relevant platform safety centre | 2 (6.06%) | 2 (7.41%) | 2 (5.26%) |
|  | Did not respond | 4 (12.12%) | 10 (37.04%) | 8 (21.05%) |
|  | Other | 13 (39.39%) | 7 (25.93%) | 5 (13.16%) |
